# COVID-19 related disruption and resilience in immunisation activities in LMICs: a rapid review

**DOI:** 10.1136/bmjopen-2023-076607

**Published:** 2024-08-06

**Authors:** Anna-Maria Hartner, Xiang Li, Katy Gaythorpe

**Affiliations:** 1 MRC Centre for Global Infectious Disease Analysis, Jameel Institute, School of Public Health, Imperial College London, London, UK; 2 Centre for Artificial Intelligence in Public Health Research, Robert Koch Institute, Wildau, Germany

**Keywords:** COVID-19, health equity, public health

## Abstract

**Abstract:**

**Objectives:**

We conducted a rapid review to determine the extent that immunisation services in low-income and middle-income countries (LMICs) were disrupted by the COVID-19 pandemic and synthesised the factors that can be used to build resilience in future.

**Design:**

Rapid review reported in accordance with the Preferred reporting for Systematic reviews and Meta-Analyses (PRISMA) guidelines.

**Data sources:**

PubMed and Web of Science were searched through 6 October 2023.

**Eligibility criteria for selecting studies:**

We included studies that focused on disruption to immunisation activities due to the COVID-19 pandemic in LMICs. Outcomes included routine vaccine coverage, supplementary immunisation activities, vaccine doses, timing of vaccination, supply chain changes, and factors contributing to disruption or resilience.

**Data extraction and synthesis:**

Two independent reviewers used standardised methods to search, screen and code studies. Quality assessment was performed using a modified version of the Critical Appraisal Skills Programme for qualitative research. Findings were summarised qualitatively.

**Results:**

Of 4978 identified studies, 85 met the eligibility criteria. Included studies showed declines in immunisation activities across LMICs related to the COVID-19 pandemic. These included reductions in achieved routine coverage, cancellation or postponement of campaigns and underimmunised cohorts. Immunisation was most disrupted in the early months of the pandemic; however, recovery varied by country, age-group and vaccine. Though many countries observed partial recovery in 2020, disruption in many countries continued into 2021. It has also been noted that clinician staff shortages and vaccine stock-outs caused by supply chain disruptions contributed to immunisation delays, but that concern over COVID-19 transmission was a leading factor. Key resiliency factors included community outreach and healthcare worker support.

**Conclusions:**

There is limited information on whether reductions in vaccination coverage or delays have persisted beyond 2021. Further research is needed to assess ongoing disruptions and identify missed vaccine cohorts.

**Strengths and limitations of this study**

- The rapid synthesis of findings through the decision to structure the paper methodologically as a rapid review allows for key insights to target missed cohorts and identify research gaps related to immunisation disruption and recovery to-date.
- We include a narrative analysis of disruption across low-income and middle-income countries; this review benefits from the inclusion of barriers, enablers and resilience to/in service provision.
- The search strategy was limited to English-language studies identified from databases PubMed and Web of Science up to 6 October 2023, meaning not all relevant research meeting inclusion criteria may have been captured.

## Introduction

 The COVID-19 pandemic began on 12 December 2019 and quickly spread globally, adding to the strain on existing healthcare provision and creating unique problems in terms of service delivery.^1^ Throughout 2020, there were disruptions to screening for cancer, maternal health services, care for chronic conditions and immunisations.^2^ This strain on health services has continued past 2020, as even those that have recovered to pre-COVID levels of visits and surveillance have to catch-up missed cohorts and delayed treatments.

Low-income and middle-income countries (LMICs) disproportionately bear the burden of vaccine preventable diseases^3^; however, globally, vaccination has seen a plateau in coverage, with zero-dose children an ongoing concern. The issue of zero-dose or underimmunised children is particularly important, as it can hint at wider heterogeneity in healthcare access which may have been exacerbated by the pandemic.^4^ It is estimated that 67 million children missed vaccinations between 2019 and 2021; of those, 48 million were zero-dose children.^5^ Furthermore, targeting zero-dose children can be more difficult as they are often in harder-to-reach areas, particularly in LMICs, where 1one in six children living in rural areas are zero-dose.^5^

Resilient healthcare systems can withstand additional and unusual strains while maintaining priority services. Yet, it is still uncertain what factors contributed to disruption or resilience in light of the COVID-19 pandemic, which was a unique test on global healthcare systems. These factors and considerations may be instrumental in preparing for future healthcare strains such as those potentially caused by other epidemics, climate change, or antimicrobial resistance. As such, understanding the key factors for disruption due to the COVID-19 pandemic is critical for future planning in order to minimise the negative consequences of disruptions.

In order to understand the current state of vaccination coverage disruption, and highlight factors contributing to resilience, we undertook a rapid review (RR) of the existing literature. This focused on LMICs as they bear the majority of burden of vaccine preventable diseases. We included studies that not only discuss the quantitative measures of disruption, such as reduced immunisation coverage and cancelled campaigns, but also more qualitative discussions of the factors contributing to disruption or characteristics of resilient systems.

## Aim and research questions

The aim of this review was to understand the extent of disruptions in vaccination coverage due to the COVID-19 pandemic and the factors that contributed to the disruption or resilience. Specifically, our research questions were:

RQ1: To what extent were immunisation services in LMICs disrupted by the COVID-19 pandemic?
RQ2: How did disruption vary by geography, demography, or socioeconomic group?
RQ3: What factors contributed to coverage disruption or resilience?

## Methods

A RR was conducted using streamlined systematic review methods and reported in accordance with the Preferred Reporting Items for Systematic Reviews and Meta-Analyses (PRISMA) guidelines.^6^ The full PRISMA checklist can be found in the online supplemental material on pages 1–5.

### Procedure

We searched PubMed and Web of science up to 6 October 2023 for studies published after 1 December 2019 in the English-language with search terms (((COVID-19) OR (SARS-CoV-2))) AND (immunisation OR vaccination) AND (disruption OR delay* OR postpon*). Studies were included if they focused on disruption to vaccination activities due to the COVID-19 pandemic in LMICs. Studies were excluded if they focused on high-income countries only, examined disruption due to other factors, that is, not related to the pandemic, or were reviews, commentaries, or modelling studies without novel data.

### Study selection, data extraction and quality assessment

Search results were imported into the Covidence (www.covidence.org) systematic review management tool where duplicates were removed. Titles and abstracts were screened by one reviewer, full-text review was completed by two reviewers with conflicts resolved through consensus.

Each study was extracted by one reviewer into a Google sheet. We extracted information on (1) last date of included data, (2) countries studied, (3) qualitative findings related to the research questions RQ1, RQ2 and RQ3 and (4) binary data on whether routine immunisation, SIAs, doses, schedule timing or supply chains were mentioned in the study. A second reviewer was consulted where there was uncertainty concerning the extracted data.

The quality of studies was assessed through a modified, nine question checklist of the Critical Appraisal Skills Programme (CASP) for qualitative studies. The 10th question, ‘how valuable is the research’, was omitted given the aim to assess quality based on binary indicators and given no studies were excluded based on determinations of value. Results were listed as N/A if not applicable for the study. All CASP results are available in the online supplemental material on pages 6–8.

### Synthesis

There were two main types of evidence to synthesise: quantitative information (ie, percentage drops in coverage achieved, doses administered, or supplementary immunisation activities (SIAs) postponed) and qualitative information on contributing factors informed by surveys or questionnaires. We grouped results by research question. Finally, we collate characteristics of the studies themselves, such as countries studied or dates of included data. For these, we have prepared summary statistics. The full list of included studies and outcomes provided are included in the online supplemental material.

### Patient and public involvement

There was no patient or public involvement in this study.

## Results

### Characteristics of studies

**Figure 1:**
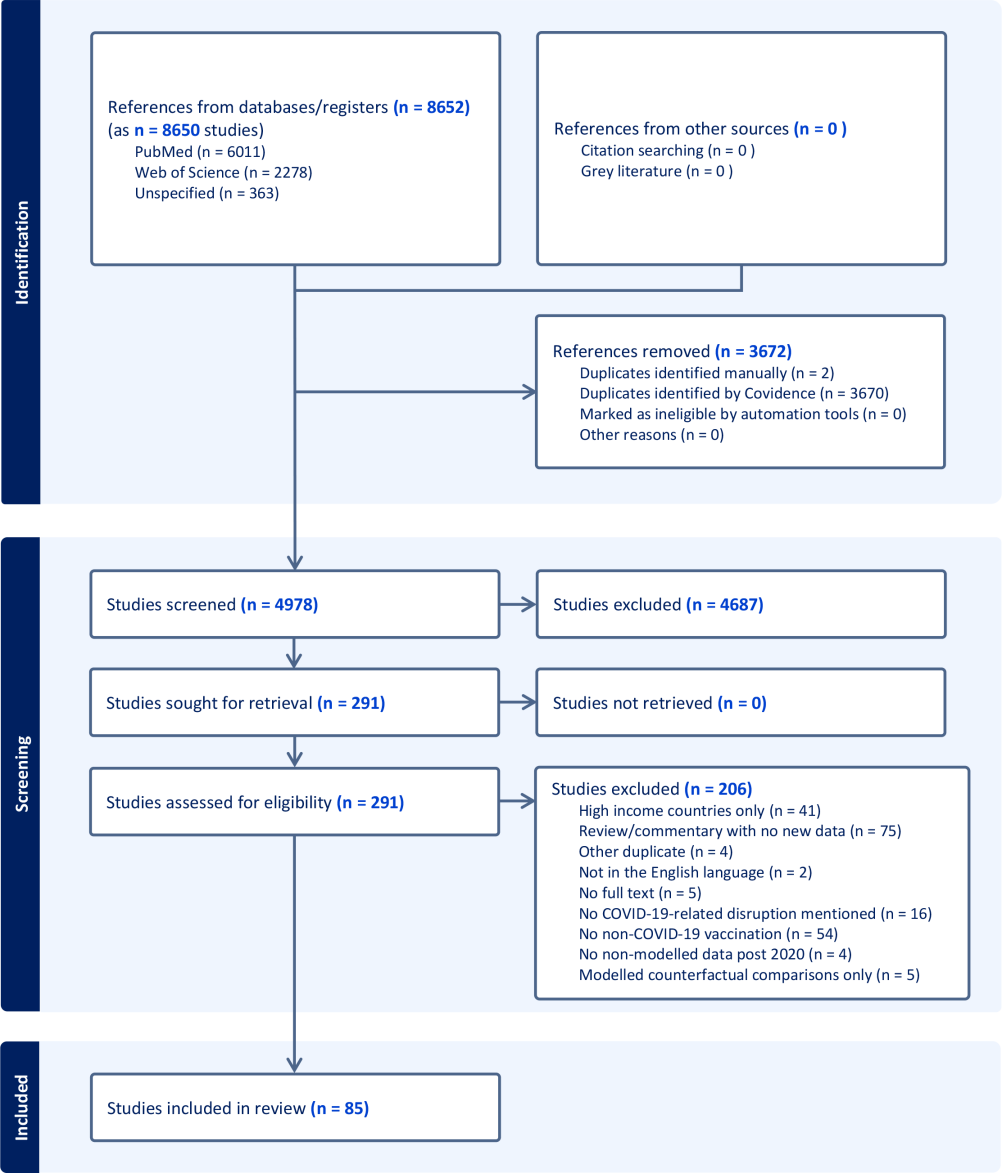
Preferred Reporting Items for Systematic Reviews and Meta-Analyses (PRISMA) flow of study selection.

We found 4978 studies where 85 met the inclusion criteria (figure 1). The majority of studies were published in either 2021 (n=30; 35.29%) or 2022 (n=32; 37.65%), though most studies only reported on data from 2020 (n=52; 61.18%). Fourteen (16.47%) studies included data during the first 6 months of 2021; a further 15 (17.64%) included data between July and December of 2021. Only four (4.71%) studies included data from 2022; the most recent of these covered data through November of 2022.

Most (n=20; 23.53%) of the studies considered multiple LMICs. Of those that only considered one country, India (n=12; 14.11%), Ethiopia (n=7; 8.24%), Pakistan (n=5; 5.88%) and Brazil (n=4; 4.71%) were the most frequently studied. The African continent was the most represented.

Most (n=57; 67.05%) studies examined the effect of the COVID-19 pandemic on routine immunisation coverage, with an additional seven (8.24%) reporting pandemic effects on SIAs. The change in the number of administered doses (n=19; 22.35%) or the timing of doses (n=15; 17.64%) was also reported by several studies; eight (9.41%) reported disruptions in the vaccine supply chain.

### Extent of disruption

We divide this section into a few main areas: supply chains and vaccine availability, the delivery of routine immunisation (as doses given, coverage and/or delays), SIAs and finally, signs of recovery (to prepandemic achieved coverage in any of the disrupted activities mentioned previously).

#### Vaccine supply

Following the declaration of COVID-19 as a pandemic, there was a reduction of vaccine sales and periods of stock-out and low availability of vaccines in some countries,^7^,^11^ though one study in Northern Nigeria found that states experienced less stock-outs in 2020 as compared with 2019.^12^ More globally, vaccine sales between April and August 2020 fell by 9.5% across 84 countries,^13^ but some losses in vaccine receipt after stock-outs were recouped by catch-up activities, such as in Uganda.^8^

#### Routine immunisation

We divide insight by WHO region or country.

In the WHO African Region, there was a varied picture of disruption. In Ethiopia, minimal disruptions were found up to August 2020.^1014^,^18^ Similarly, in the Democratic Republic of Congo (DRC), disruptions in Kinshasa were minimal up to December 2020, with one study even finding increases in diphtheria tetanus toxoid and pertussis dose 3 (DTP3) and measles-containing-vaccine first-dose (MCV1) doses administered.^19 20^ In Kenya^21^,^23^ and Burkina Faso,^24^ immunisation services were largely unaffected. Zambia saw a mixed picture, in which estimates during the first 6 months of 2020 varied month-to-month, with both disruptions and positive increases as compared with previous years; overall, however, the number of additional children missed was found to be minimal.^25^ In South Africa, however, full immunisation dropped in the first months of the pandemic, especially in April, where it dropped by 30%.^26 27^ Ghana,^28^,^30^ Nigeria,^31^ Uganda,^8 20^ Liberia,^32^ Sierra Leone^20 33^ and Somalia^11^ all saw drops in coverage in 2020, and while some countries had begun to see recovery in coverage achieved, this was not enough to compensate for missed cohorts.^20 34^

In the WHO region of the Americas, there were declines in coverage reported for the Dominican Republic, Mexico, Ecuador and Brazil. The Dominican Republic saw a drop of 10 percentage points,^35^ while vaccinations were reduced by 36% in Mexico,^36 37^ and 14% fewer doses administered were in Ecuador.^38^ In Brazil, approximately 20% of children missed vaccinations, with an 18% overall decline in doses administered in the first year of the pandemic.^39^,^41^ However, one study found no significant evidence of COVID-19 isolation measures on vaccines per child in Brazil.^42^

In the Eastern Mediterranean WHO region, drops in coverage were seen for Lebanon, Afghanistan, Jordan and Pakistan^43^,^47^ of 31%, 21%, 6%–16% and 30%–48%, respectively, over the initial stages of the pandemic. Pakistan additionally reported that as of September 2021, 18% of parents had delayed routine immunisation for their children during the pandemic; an additional 2% received no immunisations.^48^

In the South East Asian WHO region, there were significant disruptions.^49 50^ In India, six studies found substantial drops in coverage across the majority of districts (88%^51^) especially in lockdown and early in the pandemic.^52^,^56^ As a result, children born in India after COVID-19 had a 2%–10% lower probability of timely vaccination compared with earlier cohorts.^57 58^ Two studies, one conducted among the Armed Forces population in Mumbai, the other across India, found disruptions continued into 2021.^56 58^ Only one study found that the number of immunisation sessions in India increased in 2020 and 2021 compared with 2019.^59^ In Nepal and Bangladesh, the most severe disruptions were also seen earlier in the pandemic, particularly in Bangladesh, where 20%–25% of planned outreach immunisations were cancelled between April and May 2020.^60 61^ In Indonesia, one study reported that 27.4% of parents delayed compulsory immunisation in 2020.^62^

In the WHO European region, in Armenia, there were only small declines in coverage achieved.^63^ In the Western Pacific WHO Region, one study from China found that immunisation coverage dropped drastically in January 2020, but had recovered to pre-pandemic levels by June.^64^ However, a second study conducted in Beijing found pandemic effects to continue into 2021, staying below 2019 levels despite catch-up activities.^65^

Globally, there were substantial drops in routine immunisations in 2020.^9^ Overall, it was estimated that there were 31% fewer vaccine doses given.^66^ In middle-income countries, 14% of individuals delayed or missed vaccinations in the first 6 months of the pandemic,^67^ and there was a 20% increase in children who had not completed the three-dose DTP series.^68^ While disruption varied by vaccine,^69 70^ most saw the most severe declines in the 6 months of the pandemic followed by variable recovery^71^ which may affect control and elimination efforts.^72^

It was not only the total number of doses administered that was affected, but also when those doses were given. In China and India, the majority of interviewed caregivers delayed vaccination^5767 73^,^75^ and in Ecuador and Sierra Leone, this delay was worse for last doses.^33 38^

#### Supplementary immunisation activities

Overall, we found fewer studies focusing on SIAs or campaigns specifically; however, there are comprehensive records kept by the WHO campaign tracker as part of the immunisation repository.^76^ In 57 countries, SIAs were more disrupted in the early stages of the pandemic, with 57% of planned campaigns globally postponed or cancelled because of COVID-19 by May 2020.^76^ By December 2020, this had fallen to 26% and many campaigns were reinstated from July 2020 onwards. By December 2021, in 54 countries, this had fallen again to 16% of scheduled campaigns delayed or cancelled.^76^ Overall, of those campaigns disrupted between March 2020 and December 2021, 59% had been reinstated.^76^ Factors leading to postponement or cancellation of SIAs included non-pharmaceutical interventions, such as national lockdowns,^77^ and stock-outs or increased demand for general healthcare supplies.^8 78^ One study found that the national policy guidance of Mozambique and Uganda recommend the halting of campaigns in 2020.^79^ Additionally, while some SIAs had been reinstated, and there were plans for catch-up activities, there are still large missed cohorts.^9 53 72 80^

#### Recovery

Information on recovery is limited by the date ranges of the included studies, which mainly focused on 2020 and 2021. A key finding is that while there were signs of improvement in routine immunisation coverage achieved and reinstated vaccination campaigns, there was not the positive increase needed to catch-up missed cohorts, that is, a sufficient return to prepandemic levels of immunisation.^33 34 37 61 65 66 70 81^ It was also noted that pre-COVID-9 levels of coverage had not been reached in many countries by the end of 2022.^72^

### Heterogeneity in disruption

Heterogeneity in immunisation disruption was found across several factors, including geography, demography, wealth and education; these are further detailed below. Variations in the extent of disruption by antigen were similarly reported in several studies.^10 13 18 20 38 44 49 64 65 75^

#### Geographical heterogeneity

Despite significant overall decreases in immunisation in LMICs, there was geographical heterogeneity in the extent of disruption and in the regions and/or individuals affected. On a national level, several studies reported differences in the extent of disruption as a result of economic income classification^49 67 68^ by WHO region,^68 69 78^ by global burden of disease super-region,^66^ or by Gavi eligibility,^68^ with greater pandemic impact observed in LMICs compared with high-income countries, affecting the primarily African Region, the Americas and Asia. The reverse trend was seen for vaccine sales early in the pandemic (ie, April to August 2020), with high-income countries experiencing a 20% decline and low-income countries observing a 10% increase.^13^

On a subnational level, many countries observed statistically significant differences between regions, provinces or districts in regards to the change in health service utilisation,^37 51 56 59 82^ routine immunisation coverage^38 44 55^ or complete vaccination.^24^ In some countries, certain provinces reported increases in immunisation service provision or doses for some vaccines, such as in the Southern Province of Rwanda, where measles and rubella immunisation increased.^82^ Geographical heterogeneity was also observed in the subsequent recovery of services.^27 51^

While some countries reported differences in disruption between urban and rural areas, there was significant heterogeneity in the extent of disruption. One study found that the odds of immunisation in Ethiopia were higher in rural areas,^14^ while another observed greater initial declines in urban and periurban areas in South Africa, followed by recovery in these areas and declines in rural areas as the pandemic progressed.^27^ In Pakistan, lockdown affected rural areas more than urban areas.^46^ Geographical heterogeneity was also observed between Ethiopia’s hospitals and health centres, in which vaccine-related supplies were twice as likely to be affected by COVID-19 in hospitals^7 10^; in Nigeria, activities coordinated at the state level were impacted less than healthcare facilities.^12^ In China, immunisation services continued in hospitals, even when immunisation clinics were suspended.^64^

In India, children residing in ‘COVID-19 red zones’ were more likely to face immunisation disruption.^55^ Similarly, a study on polio outreach services in 33 African and Eastern Mediterranean countries found services necessary for ‘reaching their most vulnerable populations’ were partially or severely disrupted.^78^

#### Demographic heterogeneity

Few studies focused on the effects of demographic heterogeneity on COVID-19-related immunisation disruption, including factors such as gender, age, birth order or caste. Only two studies looked at differences by gender; one found greater declines in females than males, though this decline was not significant.^44^ The second, conducted in Brazil, also found no significant differences, but did find that infants were less likely to experience immunisation disruptions or delays compared with 1-year-old children.^39^ This finding was similar to two studies, conducted in Eastern India and in China, where increasing age of the child was found to be associated with immunisation delays.^74 75^ A study conducted in South-East Asia and the Western Pacific found similar results, in which early infancy was less disrupted than infancy, school-entry age and adolescent immunisation.^49^ However, greater disruption was seen among infants compared with adult/elderly immunisation.^49^ Additionally, one study in Jordan found that children older than 12 months were less likely to experience delays.^45^ Finally, one study conducted in China found firstborn children were less likely to experience delays,^75^ while another paper in India examined heterogeneity as a result of ethnicity or caste, finding lower castes had lower likelihoods of full immunisation and greater immunisation disruption, though these findings were not significant.^55^

While even fewer articles examined the demographic heterogeneity of disruption based on the characteristics of parents or caregivers, two studies stratified results by maternal or caregiver age; one finding that increasing maternal age was associated with delayed vaccination,^74^ the other finding no association.^45^ Only one study examined other contributing factors of parents, finding that women were more likely to delay vaccination for their children than men; presence of a chronic illness, prior influenza vaccination, or experience with COVID-19 diagnosis were also associated with delays in childhood vaccination.^62^

#### Socioeconomic heterogeneity

Contributors to socioeconomic heterogeneity in immunisation disruption largely included measures of household income and education. Two studies, one in Brazil and the other in India, found that missed vaccine doses were more likely in children from poorer households^39 55^; in India it was additionally found that there were greater declines in immunisation among poorer subgroups.^55^ A study in South Africa found mixed results, finding declines in full immunisation and first dose of measles greater in wealthier quintiles at the start of the pandemic, but with faster positive recovery and continued declines among poorer subgroups as the pandemic progressed.^27^ Another study in Iraq found that low socioeconomic status was associated with an increase in missed vaccine appointments.^83^ Two studies, one conducted in Iraq and one conducted in Indonesia, similarly found that the type of employment changed the odds of vaccination during the pandemic^62 83^; in Indonesia, healthcare workers (HCWs) especially were more likely to delay vaccination in their children.^62^

Only two studies, one in India and one in Iraq, focused on education, similarly finding higher probability of incomplete immunisation and greater declines in households without formal education.^55 83^

### Factors contributing to coverage disruption and resilience

We divide this section into three key areas: health system barriers, vaccine demand, and resilience.

#### Health system barriers

Many of the initial challenges in maintaining immunisation services in LMICs were the result of health system and supply barriers during the early stages of the pandemic. Many countries reported issues with vaccine supply delays or stock-outs^7^,^911 32 37 58 81 84^ and lack of personal protective equipment (PPE) for HCWs, including masks, gloves and other drugs and supplies.^7 12 31 32 51 51 53 54 84 85 87^ Disruption caused by vaccine stock-outs or supplies was found to vary by WHO region^9^ or by geographical subregion^10 31 49 87^; notably one study in Southeast Asia and the Western Pacific found vaccine stock-outs to be among the least important reasons for service provision delays.^49^ A lack of logistical support impacting routine services or outreach, such as a lack of fuel or water, was reported by three studies in the WHO African region.^31 32 84^

Similarly, HCWs availability posed a significant challenge, with countries citing difficulties due to the diversion of staff to COVID-19 response, staff illness and transportation difficulties, among others.^9 12 18 31 31 32 51 53 54 85^ One study in Kenya further reported disruption due to a HCW strike from December 2020 to January 2021.^23^ On an individual level, HCWs reported that pandemic-related stigma, stress or fears impacted service delivery,^7 31 51 54 60 85^ with some additionally reporting harassment by law enforcement or by patients themselves.^31 51^ Only one study, conducted at a tertiary health centre in Ghana, found no disruptions in vaccine supply or in HCW availability.^29^

COVID-19 lockdowns and restrictions also resulted in cancelled immunisation services, clinic closures or reduced healthcare access or services available,^7 31 32 45 46 49 55 62 67 77 83 86 88 89^ with some reporting difficulties maintaining COVID-19 prevention rules, such as social distancing, due to non-compliant patients or a lack of space.^12 31 54 84^

Competing priorities also meant some countries faced declines in funding for immunisation services or supplies, resulting in financial constraints.^12 53 87^

#### Vaccine demand and acceptance

Many of the challenges in maintaining routine immunisation services during the COVID-19 pandemic also resulted from declining vaccine demand and increasing fear or stigma surrounding COVID-19 among caregivers. Declines in vaccine demand were frequently attributed to travel barriers or difficulties in reaching immunisation services or clinics,^912 16^,^18 32 48 49 53^ COVID-19 restrictions or requirements, including testing requirements, mask requirements or lockdowns,^45 47 60 67 84 85 88^ and financial constraints.^16 53 60 67^ One study, conducted in South East Asia and the Western Pacific, reported that while affordability issues contributed to immunisation service utilisation, it was among the lowest ranked reasons.^49^ Some caregivers additionally reported low or no awareness of the availability of immunisation services, often believing clinics and hospitals were closed for routine immunisation services.^29 53 74 84^

Declines in vaccine demand due to fears of contracting COVID-19 at clinics or hospitals were pervasive, and one of the most reported causes across several studies.^912 16 18 29 32 45 47^,^49 53^ Many others reported additional fear or stigma against healthcare providers, including fears that staff might be infected by the virus.^29 32 54 60 85^ One survey of 100 caregivers at a tertiary health centre in Eastern India found that 83% of respondents agreed that ‘safety (was) more important than vaccination’.^74^ Further unspecified declines in vaccine demand were noted by several studies.^9 46 81^

Vaccine hesitancy factors were less commonly reported; misinformation and misbeliefs contributed to declines in demand in just two studies,^9 60^ while fears specifically about vaccine side effects were found in just one study in a tertiary hospital in North Ghana.^29^ One additional study in Liberia reported declines due to vaccine conspiracies, where parents believed their children would be injected with COVID-19.^32^ Only one in Ethiopia study reported fewer declines in vaccine demand as a result of COVID-19 pandemic misinformation, including that they were not susceptible to the disease, the disease was not severe, or that the pandemic did not exist.^18^

#### Resiliency

Though few papers highlighted resiliency factors or enablers to immunisation during the COVID-19 pandemic, two key focuses included community outreach to address declining vaccine demand and acceptance and the importance of improved HCW support to increase service provision. In Jordan and China, alternative arrangements for childhood vaccination (ie, outside of the standard service provision within healthcare clinics) were found to be key to maintaining immunisation demand, though in Jordan this insight was based on a survey of caregiver beliefs.^45 73^ Similarly, a community intervention highlighting the importance of maintaining timely vaccination, despite the pandemic, was crucial in Jordan, India and Ethiopia.^16 18 45 58^ Ethiopia additionally reported decreased fear of COVID-19 as an enabling factor.^16^ In India, adequate access to PPE, overcoming barriers to transportation for HCWs, community and/or family support and training on COVID-19 management were crucial to support HCWs in maintaining immunisation service provision.^85^ Similarly, proactive communication and coordination on all levels of the healthcare system was essential in Ethiopia in maintaining health system resiliency.^87^ In India, capacity building to ensure a trained workforce assisted in maintaining immunisation programmes,^58 89^ while in Nigeria, one study found the supply chain logistics to be the most important factor for maintaining immunisation services.^12^ Finally, in Zambia, community awareness through the National Immunization Campaign assisted in catching up pandemic declines.^25^

## Discussion

Despite the challenges faced by health systems during the COVID-19 pandemic, the WHO has continued to emphasise the importance of routine immunisation, noting that the last effects of immunisation declines can lead to higher burdens of disease and/or excess deaths.^90^ This review highlights the extent of disruption faced by LMICs, finding significant heterogeneity between and within regions, countries and individual demographics, but nevertheless showing declines in routine immunisation in 2020 and 2021 that had not often not recovered to pre-COVID-19 levels.

SIAs and campaigns were postponed with few regions reporting full recovery. Many LMICs rely on outreach services to reach vulnerable populations, especially where access to health clinics or services is limited.^9^ COVID-19 response efforts or mitigation strategies, including lockdowns, resulted in additional disruption to transportation services, logistical support or supplies, often hindering additional outreach activities and limiting the services that were available. This has resulted in a deepening of existing coverage inequalities, with studies noting greater disruptions among households with lower incomes, formal education or those situated in informal housing or in some regions, rural areas, emphasising the heterogeneity that existed prior to the pandemic.^91^

We used a RR format for this study, which includes some limitations. We included only two databases for the time period and only studies in English. As a result, we may be missing studies stored in other databases or in other languages. Additionally, RRs may have additional risks of bias, given the single-reviewer extraction and synthesis of findings.

The findings in this study are limited by the data available—the majority of studies used data from 2020, limiting much of our understanding of how routine immunisation services have recovered since countries lifted lockdown or other COVID-19 response policies. Our study includes only articles and does not include grey literature. Nevertheless, this study expands on the findings of a systematic review of available literature on childhood disruptions to immunisation using data from 2020, which included 39 studies and found an overall median decline of 10.8%.^92^ Additionally, our study only focuses on LMICs; this is in contrast to high-income countries, which, according to one study on 26 middle-income and high-income countries, saw considerably less missed vaccination.^67^ Our study highlights the findings through 2022 and emphasises the ongoing heterogeneity in immunisation, alongside the barriers and enablers to service provision.

Our findings also emphasise the urgency required to target individuals and cohorts who may have missed out on routine immunisation or campaigns during the COVID-19 pandemic, ensuring the barriers highlighted by staff and caretakers, including low staff or service availability, vaccine or supply stock-outs and transportation barriers are mitigated. Importantly, approaches to combat fears, misinformation or misbeliefs, including those surrounding COVID-19 transmission and risk, are critical. Though few studies touched on vaccine hesitancy, declining vaccine acceptance has become a formative issue, and additional strategies are required to prevent additional backsliding.^5^

Rebuilding immunisation services in LMICs will require a greater focus on healthcare resilience, so that the disruption caused by future epidemics or disasters on routine immunisation services is minimal, and that recovery and performance are rapid and improved through an adaptation to real-world events.^93^ Many of the countries that showed service delivery resilience during the COVID-19 pandemic highlighted the need for proactive and ongoing communication and coordination across multiple interconnected systems, especially between the community and healthcare system. One study, published in May of 2023, offers an updated framework to address the idea of epidemic-ready primary healthcare. Importantly, this framework offers solutions to many of the observed barriers found in this review, focusing on adequate training, compensation and protection for HCWs, reliable logistic and supply-chain infrastructure and linkages to the community.^94^ Given the reliance on primary healthcare and outreach systems for immunisation in LMICs, this approach may be a beneficial starting point, though notably, it will require a shift in how healthcare currently interacts with public health, alongside strong political commitment and financing.^94^ Further research will be required to understand how postpandemic disruption and recovery in immunisation services has progressed, especially in regards to vulnerable communities.

## Conclusion

This review highlights the extent and heterogeneity of immunisation disruption in LMICs as a result of the COVID-19 pandemic and the factors contributing to disruption and resilience in immunisation programmes. Given there is limited information on whether reductions in vaccination coverage or delays have persisted beyond 2021, further research is needed to assess ongoing disruptions, identify missed vaccine cohorts and examine factors contributing to resilience. Furthermore, these findings highlight the need for immunisation programmes to provide support for HCWs and proactive communication within the health system and with the wider community to ensure the effect of future disasters on vulnerable communities is minimal.

## supplementary material

10.1136/bmjopen-2023-076607 online supplemental file 1

## Data Availability

All data relevant to the study are included in the article or uploaded as supplementary information.
