# Supplementary material for: COVID-19 related disruption and resilience in immunisation activities in LMICs: a rapid review
Source: BMJ Open. 2024 Aug 6;14(8):e076607. doi: 10.1136/bmjopen-2023-076607 (PMC11331918; doi:10.1136/bmjopen-2023-076607)
Supplement: online supplemental file 1 [file bmjopen-14-8-s001.pdf]

Supplementary Index:
COVID-19-related disruption and resiliency in immunisation activities in LMICs: a rapid review

Anna-Maria Hartner, Xiang Li, Katy Gaythorpe

July 15, 2024

Search Strategy

We searched PubMed and Web of science on 6th October 2023 for studies published after 1st December 2019 in English. Our full list of search terms was as follows: (((COVID-19) OR (SARS-CoV-2))) AND (immunisation OR vaccination) AND (disruption OR delay* OR postpon*).

PRISMA Checklist

The PRISMA checklist is referenced in the main text on page 3.

Section and Topic	Item	Checklist item	Location where item is reported
TITLE			
Title	1	Identify the report as a systematic review.	Title identifies study as a rapid review (page 1).
ABSTRACT			
Abstract	2	See the PRISMA 2020 for Abstracts checklist.	Abstract follows BMJ Open guidelines (pages 1-2).
INTRODUCTION			
Rationale	3	Describe the rationale for the review in the context of existing knowledge.	Introduction (pages 2-3).
Objectives	4	Provide an explicit statement of the objective(s) or question(s) the review addresses.	Aim and research questions (page 3).
METHODS			
Eligibility criteria	5	Specify the inclusion and exclusion criteria for the review and how studies were grouped for the syntheses.	Methods: procedure (page 3).
Information sources	6	Specify all databases, registers, websites, organisations, reference lists and other sources searched or consulted to identify studies. Specify the date when each source was last searched or consulted.	Methods: procedure (page 3).
Search strategy	7	Present the full search strategies for all databases, registers and websites, including any filters and limits used.	Methods: procedure (page 3) and supplementary index (page 1).
Selection process	8	Specify the methods used to decide whether a study met the inclusion criteria of the review, including how many reviewers screened each record and each report retrieved, whether they worked independently, and if applicable, details of automation tools used in the process.	Methods: Study selection, data extraction and quality assessment (pages 3-4).
Data collection process	9	Specify the methods used to collect data from reports, including how many reviewers collected data from each report, whether they worked independently, any processes for obtaining or confirming data from study investigators, and if applicable, details of automation tools used in the process.	Methods: Study selection, data extraction and quality assessment (pages 3-4).

Section and Topic	Item	Checklist item	Location where item is reported
Data items	10a	List and define all outcomes for which data were sought. Specify whether all results that were compatible with each outcome domain in each study were sought (e.g. for all measures, time points, analyses), and if not, the methods used to decide which results to collect.	Methods: Study selection, data extraction and quality assessment (pages 3-4).
	10b	List and define all other variables for which data were sought (e.g. participant and intervention characteristics, funding sources). Describe any assumptions made about any missing or unclear information.	Methods: Study selection, data extraction and quality assessment (pages 3-4).
Study risk of bias assessment	11	Specify the methods used to assess risk of bias in the included studies, including details of the tool(s) used, how many reviewers assessed each study and whether they worked independently, and if applicable, details of automation tools used in the process.	Methods: Study selection, data extraction and quality assessment (pages 3-4).
Effect measures	12	Specify for each outcome the effect measure(s) (e.g. risk ratio, mean difference) used in the synthesis or presentation of results.	N/A; narrative synthesis only.
Synthesis methods	13a	Describe the processes used to decide which studies were eligible for each synthesis (e.g. tabulating the study intervention characteristics and comparing against the planned groups for each synthesis (item 5)).	All studies meeting inclusion criteria were synthesized.
	13b	Describe any methods required to prepare the data for presentation or synthesis, such as handling of missing summary statistics, or data conversions.	N/A; narrative synthesis only.
	13c	Describe any methods used to tabulate or visually display results of individual studies and syntheses.	N/A
	13d	Describe any methods used to synthesize results and provide a rationale for the choice(s). If meta-analysis was performed, describe the model(s), method(s) to identify the presence and extent of statistical heterogeneity, and software package(s) used.	Methods: Synthesis (page 4).
	13e	Describe any methods used to explore possible causes of heterogeneity among study results (e.g. subgroup analysis, meta-regression).	N/A; narrative synthesis only.
	13f	Describe any sensitivity analyses conducted to assess robustness of the synthesized results.	N/A; narrative synthesis only.
Reporting bias assessment	14	Describe any methods used to assess risk of bias due to missing results in a synthesis (arising from reporting biases).	N/A; limitations in discussion (pages 9-10).
Certainty assessment	15	Describe any methods used to assess certainty (or confidence) in the body of evidence for an outcome.	N/A; narrative synthesis only.

Section and Topic	Item	Checklist item	Location where item is reported
RESULTS			
	16a	Describe the results of the search and selection process, from the number of records identified in the search to the number of studies included in the review, ideally using a flow diagram.	Results: Characteristics of studies (page 4).
Study selection	16b	Cite studies that might appear to meet the inclusion criteria, but which were excluded, and explain why they were excluded.	Results: Characteristics of studies (page 4).
Study characteristics	17	Cite each included study and present its characteristics.	Results: Characteristics of studies (page 4); all studies are cited throughout.
Risk of bias in studies	18	Present assessments of risk of bias for each included study.	Supplementary Index: pages 9-10
Results of individual studies	19	For all outcomes, present, for each study: (a) summary statistics for each group (where appropriate) and (b) an effect estimate and its precision (e.g. confidence/credible interval), ideally using structured tables or plots.	N/A; narrative synthesis only
	20a	For each synthesis, briefly summarise the characteristics and risk of bias among contributing studies.	Results; bias in discussion (pages 4-10).
	20b	Present results of all statistical syntheses conducted. If meta-analysis was done, present for each the summary estimate and its precision (e.g. confidence/credible interval) and measures of statistical heterogeneity. If comparing groups, describe the direction of the effect.	N/A; narrative synthesis only.
Results of syntheses	20c	Present results of all investigations of possible causes of heterogeneity among study results.	N/A; narrative synthesis only.
	20d	Present results of all sensitivity analyses conducted to assess the robustness of the synthesized results.	N/A; narrative synthesis only.
Reporting biases	21	Present assessments of risk of bias due to missing results (arising from reporting biases) for each synthesis assessed.	Discussion (pages 9-10).
Certainty of evidence	22	Present assessments of certainty (or confidence) in the body of evidence for each outcome assessed.	Discussion (pages 9-10)
DISCUSSION			
	23a	Provide a general interpretation of the results in the context of other evidence.	Discussion (pages 9-10)
	23b	Discuss any limitations of the evidence included in the review.	Discussion (pages 9-10)
	23c	Discuss any limitations of the review processes used.	Discussion (pages 9-10)

Section and Topic	Item	Checklist item	Location where item is reported
Discussion			
	23d	Discuss implications of the results for practice, policy, and future research.	Discussion (pages 9-10)
OTHER INFORMATION			
Registration and protocol	24a	Provide registration information for the review, including register name and registration number, or state that the review was not registered.	Review was not registered.
	24b	Indicate where the review protocol can be accessed, or state that a protocol was not prepared.	Protocol was not prepared.
	24c	Describe and explain any amendments to information provided at registration or in the protocol.	N/A
Support	25	Describe sources of financial or non-financial support for the review, and the role of the funders or sponsors in the review.	Acknowledgments
Competing interests	26	Declare any competing interests of review authors.	Acknowledgments
Availability of data, code and other materials	27	Report which of the following are publicly available and where they can be found: template data collection forms; data extracted from included studies; data used for all analyses; analytic code; any other materials used in the review.	Articles used in narrative synthesis are publicly available and given in citations.

Table 1: Preferred Reporting Items for Systematic reviews and Meta-Analyses (PRISMA) Checklist

Critical Appraisal Skills Program Quality Assessment

Study	Aims stated?	Appropriate methods?	Appropriate methods for aims?	Appropriate recruitment strategy for aims?	Data collected in way that addressed research question?	Relationship between researcher and participants?	Ethical consideration?	Rigorous Data Analysis?	Clear findings?
Hou et. al., 2021	Yes	Yes	Yes	Yes	Yes	Primary Data	Yes	Yes	Yes
Bose et al., 2022	Yes	Yes	Yes	N/A	Yes	Secondary Data	Yes	Yes	Yes
Mansour et al., 2021	Yes	Yes	Yes	Yes	Yes	Primary and Secondary Data	Yes	No	Yes
Wanyana et. al., 2021	Yes	Yes	Yes	N/A	Yes	Secondary Data	Yes	No	Yes
Saso et. al., 2020	Yes	Yes	Yes	Yes	Yes	Primary Data	Yes	Yes	Yes
Carter et. al., 2022	Yes	Yes	Yes	Yes	Yes	Primary Data	Yes	Yes	Yes
Shapiro et. al., 2022	Yes	Yes	Yes	Yes	Yes	Primary and Secondary Data	Yes	Yes	Yes
Desta et. al., 2021	Yes	Yes	Yes	Yes	Yes	Secondary Data	No	Yes	Yes
Jensen et. al., 2020	Yes	Yes	Yes	Yes	Yes	Secondary Data	Yes	Yes	Yes
Silveira et. al., 2021	Yes	Yes	Yes	Yes	Yes	Secondary Data	Yes	No	Yes
Harris et. al., 2021	Yes	Yes	No	No	Yes	Primary Data	Yes	Yes	Yes
Chandir et. al., 2020	Yes	Yes	Yes	Yes	Yes	Secondary Data	Yes	Yes	Yes
Shapira et. al., 2021	Yes	Yes	Yes	N/A	Yes	Secondary Data	Yes	Yes	Yes
Abid et. al., 2022	Yes	Yes	Yes	N/A	Yes	Secondary Data	Yes	No	Yes
Khan et. al., 2021	Yes	Yes	Yes	N/A	Yes	Secondary Data	No	Yes	Yes
Zeitouny et. al., 2021	Yes	Yes	Yes	N/A	Yes	Secondary Data	Yes	Yes	Yes
Cabral et. al., 2021	Yes	Yes	No	N/A	Yes	Secondary Data	Yes	Yes	Yes
Singh et. al., 2021	Yes	Yes	Yes	Yes	Yes	Primary Data	Yes	Yes	Yes
Patel et. al., 2022	Yes	Yes	Yes	Yes	Yes	Primary Data	Yes	Yes	Yes
Bekele et. al., 2022	Yes	Yes	Yes	Yes	Yes	Primary and Secondary Data	Yes	Yes	Yes
Shet et. al., 2021	Yes	Yes	Yes	Yes	Yes	Primary Data	Yes	No	Yes
Nguyenet. al., 2021	Yes	Yes	Yes	Yes	Yes	Primary and Secondary Data	Yes	Yes	Yes
Mishra et. al., 2023	Yes	Yes	Yes	Yes	Yes	Primary Data	Yes	No	Yes
Wang et. al., 2022	Yes	Yes	Yes	Yes	Yes	Primary Data	Yes	Yes	Yes
Jain et. al., 2021	Yes	Yes	Yes	Yes	Yes	Secondary Data	Yes	Yes	Yes
Avula et. al., 2022	Yes	Yes	Yes	Yes	Yes	Primary and Secondary Data	Yes	No	Yes
Nigus et. al., 2020	No	N/A	N/A	N/A	N/A	Secondary Data	No	No	Yes
Assefa et. al., 2021	Yes	Yes	Yes	Yes	Yes	Primary Data	Yes	Yes	Yes
Kawakatsu et. al., 2023	Yes	Yes	Yes	N/A	Yes	Secondary Data	Yes	Yes	Yes
Adelekan et. al., 2021	Yes	Yes	Yes	Yes	Yes	Primary Data	Yes	Yes	Yes
de Oliveira et. al., 2022	Yes	Yes	Yes	N/A	Yes	Secondary Data	No	Yes	Yes
Shet et. al., 2022	Yes	Yes	Yes	N/A	Yes	Secondary Data	Yes	No	Yes
Muhoza et. al., 2021	No	Yes	N/A	N/A	N/A	Secondary Data	No	N/A	Yes
Colomé-Hidalgo et. al., 2022	Yes	Yes	Yes	N/A	Yes	Secondary Data	No	Yes	Yes
Santos et. al., 2021	Yes	Yes	Yes	N/A	Yes	Secondary Data	Yes	Yes	Yes
Doubova et. al., 2021	Yes	Yes	Yes	N/A	Yes	Secondary Data	Yes	Yes	Yes
Burt et. al., 2021	Yes	Yes	Yes	N/A	Yes	Secondary Data	Yes	Yes	Yes
Hategeka et. al., 2021	Yes	Yes	Yes	N/A	Yes	Secondary Data	Yes	Yes	Yes
Causey et. al., 2021	Yes	Yes	Yes	N/A	Yes	Secondary Data	No	Yes	Yes
Alves et. al., 2021	Yes	Yes	Yes	N/A	Yes	Secondary Data	Yes	Yes	Yes
Abu-Rish et. al., 2022	Yes	Yes	Yes	Yes	Yes	Primary and Secondary Data	Yes	Yes	Yes
Babalola et. al., 2022	Yes	Yes	Yes	Yes	Yes	Primary Data	Yes	Yes	Yes
Evanset. al., 2022	Yes	Yes	Yes	N/A	Yes	Secondary Data	No	Yes	Yes
Khatiwada et. al., 2021	Yes	Yes	Yes	Yes	Yes	Primary Data	Yes	Yes	Yes
Suárez-Rodríguez et. al., 2022	Yes	Yes	Yes	N/A	Yes	Secondary Data	Yes	Yes	Yes
Shaikh et. al., 2021	Yes	Yes	Yes	N/A	Yes	Secondary Data	No	Yes	Yes
Rahman et. al., 2021	Yes	Yes	Yes	N/A	Yes	Secondary Data	No	No	Yes

Study	Aims stated?	Appropriate methods?	Appropriate methods for aims?	Appropriate recruitment strategy for aims?	Data collected in way that addressed research question?	Relationship between researcher and participants?	Ethical consideration?	Rigorous Data Analysis?	Clear findings?
Khan et. al., 2022	Yes	Yes	Yes	Yes	Yes	Primary Data	Yes	No	Yes
Bimpong et. al., 2021	Yes	Yes	Yes	Yes	Yes	Primary and Secondary Data	Yes	Yes	Yes
Rana et. al., 2021	No	N/A	No	N/A	Yes	Secondary Data	No	No	Yes
Powelson et. al., 2022	Yes	Yes	Yes	Yes	Yes	Primary Data	Yes	Yes	Yes
Wambua et. al., 2022	Yes	Yes	Yes	N/A	Yes	Secondary Data	Yes	Yes	Yes
Mariani et. al., 2022	Yes	Yes	Yes	N/A	Yes	Secondary Data	Yes	No	Yes
Summan et. al., 2023	Yes	Yes	Yes	N/A	Yes	Secondary Data	No	Yes	Yes
Thsehla et. al., 2023	Yes	Yes	Yes	N/A	Yes	Secondary Data	Yes	Yes	Yes
Adilo et. al., 2022	Yes	Yes	Yes	Yes	Yes	Primary and Secondary Data	Yes	Yes	Yes
Lucinde et. al., 2023	Yes	Yes	Yes	Yes	Yes	Primary Data	Yes	Yes	Yes
Connolly et. al., 2022	Yes	Yes	Yes	N/A	Yes	Secondary Data	Yes	Yes	Yes
Burkholder et. al., 2021	Yes	Yes	Yes	N/A	Yes	Secondary Data	No	Yes	Yes
Mbaeyi et. al., 2021	No	Yes	N/A	N/A	Yes	Secondary Data	No	Yes	Yes
Kiarie et. al., 2022	Yes	Yes	Yes	N/A	Yes	Secondary Data	Yes	Yes	Yes
Doubova et. al., 2022	Yes	Yes	Yes	N/A	Yes	Secondary Data	Yes	Yes	Yes
Ho et. al., 2022	Yes	Yes	Yes	N/A	Yes	Secondary Data	Yes	No	Yes
Melkonyan et. al., 2022	Yes	Yes	Yes	N/A	Yes	Secondary Data	Yes	No	Yes
Kissi et. al., 2022	Yes	Yes	Yes	Yes	Yes	Primary Data	Yes	No	No
Minta et. al., 2022	No	Yes	No	N/A	Yes	Secondary Data	No	Yes	Yes
Plotkin et. al., 2022	Yes	Yes	Yes	N/A	Yes	Secondary Data	Yes	Yes	Yes
Cooper et. al., 2023	Yes	Yes	Yes	Yes	Yes	Primary and Secondary Data	Yes	Yes	Yes
Kasonia et. al., 2023	Yes	Yes	Yes	Yes	Yes	Primary and Secondary Data	Yes	Yes	Yes
Winter et. al., 2023	Yes	Yes	Yes	N/A	Yes	Secondary Data	Yes	Yes	Yes
Fahriani et. al., 2021	Yes	Yes	Yes	Yes	Yes	Primary Data	Yes	Yes	Yes
Orey et. al., 2023	Yes	Yes	Yes	N/A	Yes	Secondary Data	Yes	No	No
Sucharitha et. al., 2022	Yes	Yes	Yes	Yes	Yes	Primary Data	Yes	Yes	Yes
Manzoor et. al., 2022	Yes	Yes	Yes	Yes	Yes	Primary Data	Yes	No	Yes
Tawar et. al., 2022	Yes	No	Yes	Yes	Yes	Primary and Secondary Data	No	No	No
Wu et. al., 2020	Yes	Yes	Yes	N/A	Yes	Secondary Data	No	Yes	Yes
Owais et. al., 2023	Yes	Yes	Yes	N/A	Yes	Secondary Data	No	No	No
Rodo et. al., 2022	Yes	Yes	Yes	Yes	Yes	Primary and Secondary Data	Yes	Yes	Yes
Chakrabarti et. al., 2023	Yes	Yes	No	N/A	Yes	Secondary Data	Yes	No	Yes
Berhane et. al., 2023	Yes	Yes	Yes	Yes	Yes	Primary and Secondary Data	Yes	Yes	Yes
Zeidan et. al., 2023	Yes	Yes	Yes	Yes	Yes	Primary and Secondary Data	No	No	Yes
Aigbogun et. al., 2023	Yes	Yes	Yes	Yes	Yes	Primary and Secondary Data	Yes	Yes	No
Ji et. al., 2023	Yes	Yes	Yes	Yes	Yes	Primary Data	Yes	Yes	Yes
Endehabtu et. al., 2023	Yes	Yes	Yes	Yes	Yes	Primary Data	Yes	No	Yes
Sharma et. al., 2023	Yes	No	Yes	N/A	Yes	Secondary Data	Yes	No	No

Table 2: Modified Critical Appraisal Skills Program Quality Assessment for Qualitative Studies

Additional Supplementary Tables

OVERVIEW		OUTCOMES				
**Study**	**Countries**	**Routine coverage**	**SIA**	**Doses**	**Dose timing**	**Supply chain**
Hou et. al., 2021	China				TRUE	
Bose et al., 2022	Nepal		TRUE			
Mansour et al., 2021	Lebanon	TRUE		TRUE		
Wanyana et. al., 2021	Rwanda					
Saso et. al., 2020	Multiple	TRUE				
Carter et. al., 2022	Ethiopia	TRUE				
Shapiro et. al., 2022	Multiple	TRUE				
Desta et. al., 2021	Ethiopia	TRUE				
Jensen et. al., 2020	South Africa	TRUE				
Silveira et. al., 2021	Brazil	TRUE				
Harris et. al., 2021	SEAR/WPR	TRUE				
Chandir et. al., 2020	Pakistan	TRUE			TRUE	
Shapira et. al., 2021	Subsaharan Africa	TRUE		TRUE	TRUE	
Abid et. al., 2022	Afghanistan	TRUE				
Khan et. al., 2021	India			TRUE		
Zeitouny et. al., 2021	Multiple			TRUE		TRUE
Cabral et. al., 2021	Brazil and Portugal					
Singh et. al., 2021	Nepal	TRUE				
Patel et. al., 2022	India	TRUE				TRUE
Bekele et. al., 2022	Ethiopia					
Shet et. al., 2021	India	TRUE				
Nguyenet. al., 2021	India	TRUE				
Mishra et. al., 2023	India				TRUE	
Wang et. al., 2022	China				TRUE	
Jain et. al., 2021	India	TRUE		TRUE	TRUE	
Avula et. al., 2022	India					
Nigus et. al., 2020	Ethiopia					
Assefa et. al., 2021	Multiple	TRUE		TRUE		
Kawakatsu et. al., 2023	Ghana	TRUE				
Adelekan et. al., 2021	Nigeria	TRUE		TRUE		
de Oliveira et. al., 2022	Brazil			TRUE		
Shet et. al., 2022	Multiple	TRUE	TRUE			
Muhoza et. al., 2021	Multiple	TRUE				
Colomé-Hidalgo et. al., 2022	Dominican Republic	TRUE				
Santos et. al., 2021	Brazil	TRUE				
Doubova et. al., 2021	Mexico	TRUE				
Burt et. al., 2021	Uganda	TRUE	TRUE			TRUE
Hategeka et. al., 2021	DRC	TRUE				
Causey et. al., 2021	Multiple	TRUE				
Alves et. al., 2021	Brazil	TRUE				
Abu-Rish et. al., 2022	Jordan	TRUE			TRUE	

Babalola et. al., 2022	Liberia	TRUE				TRUE
Evanset. al., 2022	Multiple	TRUE				
Khatiwada et. al., 2021	Nepal				TRUE	TRUE
Suárez-Rodríguez et. al., 2022	Ecuador	TRUE		TRUE		
Shaikh et. al., 2021	Multiple	TRUE				
Rahman et. al., 2021	Pakistan	TRUE				
Khan et. al., 2022	Pakistan				TRUE	
Bimpong et. al., 2021	Ghana	TRUE				
Rana et. al., 2021	Bangladesh	TRUE				
Powelson et. al., 2022	Mozambique	TRUE		TRUE	TRUE	
Wambua et. al., 2022	Kenya	TRUE				
Mariani et. al., 2022	Sierra Leone			TRUE		
Summan et. al., 2023	India	TRUE			TRUE	
Thsehla et. al., 2023	South Africa	TRUE		TRUE		
Adilo et. al., 2022	Ethiopia					TRUE
Lucinde et. al., 2023	Kenya	TRUE				
Connolly et. al., 2022	Multiple	TRUE				
Burkholder et. al., 2021	Multiple	TRUE	TRUE			TRUE
Mbaeyi et. al., 2021	Pakistan					
Kiarie et. al., 2022	Kenya			TRUE		
Doubova et. al., 2022	Mexico	TRUE				
Ho et. al., 2022	Multiple		TRUE			
Melkonyan et. al., 2022	Armenia	TRUE				
Kissi et. al., 2022	Ghana	TRUE				
Minta et. al., 2022	Multiple	TRUE	TRUE			
Plotkin et. al., 2022	Multiple	TRUE	TRUE			
Cooper et. al., 2023	Burkina Faso	TRUE				
Kasonia et. al., 2023	Multiple			TRUE		
Winter et. al., 2023	Zambia	TRUE				
Fahriani et. al., 2021	Indonesia				TRUE	
Orey et. al., 2023	Somalia	TRUE				
Sucharitha et. al., 2022	India			TRUE		
Manzoor et. al., 2022	Pakistan				TRUE	
Tawar et. al., 2022	India	TRUE		TRUE		
Wu et. al., 2020	China	TRUE		TRUE		
Owais et. al., 2023	South Asia	TRUE				
Rodo et. al., 2022	Multiple	TRUE				
Chakrabarti et. al., 2023	India			TRUE		
Berhane et. al., 2023	Ethiopia			TRUE		
Zeidan et. al., 2023	Iraq				TRUE	
Aigbogun et. al., 2023	Nigeria	TRUE				TRUE
Ji et. al., 2023	China	TRUE			TRUE	
Endehabtu et. al., 2023	Ethiopia					TRUE
Sharma et. al., 2023	India					

Table 3: Overview of countries examined and outcomes reported (routine coverage, supplementary immunisation campaigns (SIAs), dose timing, or supply chain disruptions) by included papers.
