# Supplementary material for: COVID-19 related disruption and resilience in immunisation activities in LMICs: a rapid review
Source: BMJ Open. 2024 Aug 6;14(8):e076607. doi: 10.1136/bmjopen-2023-076607 (PMC11331918; doi:10.1136/bmjopen-2023-076607)
Supplement: Supplementary file 2 [file bmjopen-2023-076607.reviewer_comments.pdf]

PEER REVIEW HISTORY

BMJ Open publishes all reviews undertaken for accepted manuscripts. Reviewers are asked to complete a checklist review form (**http://bmjopen.bmj.com/site/about/resources/checklist.pdf**) and are provided with free text boxes to elaborate on their assessment. These free text comments are reproduced below.

ARTICLE DETAILS

**TITLE (PROVISIONAL)**	COVID-19-related disruption and resilience in immunisation activities in LMICs: a rapid review
**AUTHORS**	Hartner, Anna-Maria; Li, Xiang; Gaythorpe, Katy

VERSION 1 – REVIEW

**REVIEWER**	Rao, Chythra R.<br>Manipal University
**REVIEW RETURNED**	04-Jul-2023

**GENERAL COMMENTS**	3.1: Procedure<br>What search terms were used for LMICs?<br>Fig 1: The exact PRISMA flow chart needs to be followed.(screening, eligibility, inclusion) The reasons for exclusion also needs to be mentioned.<br>4.2: Extent of disruption<br>What does signs of recovery refer to? Child health?covid infection? Or immunization activity?<br>4.2.3 Supplementary immunisation activities<br>Factors leading to postponement or cancellation of SIAs included non-pharmaceutical interventions [62] – please provide details on non- pharmaceutical interventions leading to postponement or cancellation of SIAs<br>4.2.4 Recovery<br>there was not the positive increase needed to catch up missed cohorts [27, 51, 55, 49, 30, 65, 26]. – how was positive increase defined?<br>PRISMA checklist to be enclosed

**REVIEWER**	Al-Kuwari, Mohamed Ghaith<br>Primary Health Care Corporation, Family & Community Medicine
**REVIEW RETURNED**	18-Nov-2023

**GENERAL COMMENTS**	This systematic review addresses important public health impact of the recent pandemic. It focuses on LMICs. I whish to see comparison of that to HICs is there is any significant difference?<br><br>The manuscript is clear, well written, and easy to read.

**REVIEWER**	Nabia, Sarah<br>Johns Hopkins University, International Health
**REVIEW RETURNED**	10-Mar-2024

**GENERAL COMMENTS**	This is an important topic to be studied. Thank you to the authors for attempting this research question. Below are the comments:

	Comments on methods:<br>• The Cochrane guidance on rapid review states at least 2 databases should be searched - this review only looked at 1. This severely limits the data used in this review. The authors should consider adding more databases. At this point, this is inadequate to be called a rapid review. (Garritty C, Hamel C, Trivella M, Gartlehner G, Nussbaumer-Streit B, Devane D, Kamel C, Griebler U, King VJ; Cochrane Rapid Reviews Methods Group. Updated recommendations for the Cochrane rapid review methods guidance for rapid reviews of effectiveness. BMJ. 2024 Feb 6;384:e076335. doi: 10.1136/bmj-2023-076335. PMID: 38320771.)<br>• Quality assessment of the papers is necessary - this is also reflected in the best practices for rapid review. Not attempting a quality assessment leaves the readers with very little idea of the quality of evidence they are reading. It is okay for the authors to be flexible with standards of quality (eg all included papers don't need to meet all criteria, and they can instead place importance on what they think are more important criteria). Irrespective, its essential to do a quality assessment of the papers.<br><br>PRISMA : Please include reason for excluding papers and the corresponding number of papers for each reason.<br><br>4.2.1 vaccine supply: The authors mention vaccine sales fell by 9.5% and some losses were recouped by catch up activities. This statement is inadequate. How many and which countries were considered in this? How did you arrive at the 9.5%? Some quantification / justification is required where the authors say 'some losses were recouped'. A table or graphic to support this statement would be useful.<br><br>4.2.2 Routine immunisation: The authors report multiple indicators here - coverage, doses given, missed vaccination, etc. This is confusing to read. Suggestion to organize in a table with the following column headings: paper, country, and then list all possible indicators; then report all relevant indicators for the paper.<br><br>4.3.1 Geographic heterogeneity: While the takeaway messages on the heterogeneity is clear, this section severely lacks justification in terms of numbers. For every heterogeneity that authors discuss, the statistics they are using / referring to should also be clearly stated.<br><br>Finally, this paper needs a conclusion.<br><br>Overall, the paper needs proof reading and correction of grammatical errors.

VERSION 1 – AUTHOR RESPONSE

Reviewer: 1
3.1: Procedure
What search terms were used for LMICs?

Thank you for your question. We did not use search terms to filter by LMICs, but rather excluded all high-income countries during the abstract/title screening and full-text review.

Fig 1: The exact PRISMA flow chart needs to be followed.(screening, eligibility, inclusion) The reasons for exclusion also needs to be mentioned.

Thank you for the feedback. We have updated the PRIMA flow chart and included the reasons for exclusion.

4.2: Extent of disruption
What does signs of recovery refer to? Child health?covid infection? Or immunization activity?

Thank you for your question. In this instance, signs of recovery refers to recovery in any of the areas of potential disruption mentioned prior, i.e. supply chains and vaccine availability, delivery of routine immunisation, and/or supplementary immunisation activities. We have clarified this in the opening statement of this section.

4.2.3 Supplementary immunisation activities
Factors leading to postponement or cancellation of SIAs included non-pharmaceutical interventions [62] – please provide details on non- pharmaceutical interventions leading to postponement or cancellation of SIAs

We have updated this section to specify that NPI in this instance included nationwide lockdowns.

4.2.4 Recovery
there was not the positive increase needed to catch up missed cohorts [27, 51, 55, 49, 30, 65, 26]. – how was positive increase defined?

A positive increase was defined as any increase in immunisation coverage or activities towards pre-pandemic levels, thus mitigating the disruption in cohorts as a result of the COVID-19 pandemic. This has been clarified in this section.

PRISMA checklist to be enclosed

Thank you, we have included the PRISMA checklist in the supplementary materials.
Reviewer: 2
Dr. Mohamed Ghaith Al-Kuwari, Primary Health Care Corporation
Comments to the Author:
This systematic review addresses important public health impact of the recent pandemic. It focuses on LMICs. I whish to see comparison of that to HICs is there is any significant difference?

Thank you. High-income countries did not see the extent of disruption in immunisations that low- and middle-income countries did as a result of the COVID-19 pandemic. This is further discussed in our results section, under geographic heterogeneity.

The manuscript is clear, well written, and easy to read.

Thank you for your comment.
Reviewer: 3
Dr. Sarah Nabia, Johns Hopkins University
Comments to the Author:

This is an important topic to be studied. Thank you to the authors for attempting this research question. Below are the comments:

Comments on methods:
• The Cochrane guidance on rapid review states at least 2 databases should be searched - this review only looked at 1. This severely limits the data used in this review. The authors should consider adding more databases. At this point, this is inadequate to be called a rapid review. (Garritty C, Hamel C, Trivella M, Gartlehner G, Nussbaumer-Streit B, Devane D, Kamel C, Griebler U, King VJ; Cochrane Rapid Reviews Methods Group. Updated recommendations for the Cochrane rapid review methods guidance for rapid reviews of effectiveness. BMJ. 2024 Feb 6;384:e076335. doi: 10.1136/bmj-2023-076335. PMID: 38320771.)

Thank you, we have expanded the dates of our search to October 6th, 2023, and added an additional database. This led to an additional 19 studies included in our review.

• Quality assessment of the papers is necessary - this is also reflected in the best practices for rapid review. Not attempting a quality assessment leaves the readers with very little idea of the quality of evidence they are reading. It is okay for the authors to be flexible with standards of quality (eg all included papers don't need to meet all criteria, and they can instead place importance on what they think are more important criteria). Irrespective, its essential to do a quality assessment of the papers.

Thank you, we added a quality assessment using a modified version of the Critical Appraisal Skills Programme (CASP) for qualitative research. These results are now included in our supplementary index.

PRISMA : Please include reason for excluding papers and the corresponding number of papers for each reason.

We have updated the PRISMA chart to include the reasons for exclusion.

4.2.1 vaccine supply: The authors mention vaccine sales fell by 9.5% and some losses were recouped by catch up activities. This statement is inadequate. How many and which countries were considered in this? How did you arrive at the 9.5%? Some quantification / justification is required where the authors say 'some losses were recouped'. A table or graphic to support this statement would be useful.

Thank you for your comment. We've provided additional context for this statement in this section. The 9.5% statistic was calculated by Zeitouny et al., as cited. Quantifying the recuperation of losses isn't feasible, as the statement originates from a separate research article focused on Uganda, where authors discussed catch-up activities following an extended stockout period. Considering these circumstances, it wouldn't be suitable to include a table or graphic, as would merely be a reproduction from another research article.

4.2.2 Routine immunisation: The authors report multiple indicators here - coverage, doses given, missed vaccination, etc. This is confusing to read. Suggestion to organize in a table with the following column headings: paper, country, and then list all possible indicators; then report all relevant indicators for the paper.

Thank you for your comment. We have included an additional table in the supplementary index.

4.3.1 Geographic heterogeneity: While the takeaway messages on the heterogeneity is clear, this section severely lacks justification in terms of numbers. For every heterogeneity that authors discuss, the statistics they are using / referring to should also be clearly stated.

Thank you for your comment. The aims of this article were to rapidly assess whether any differences in the extent of immunisation disruption existed across geographic regions and/or within-country regions. Given the differences in reporting, statistical methods, administrative boundaries, and that many results were not often quantified in included studies, summary statistics are not feasible to calculate beyond this.

Finally, this paper needs a conclusion.

We have added a conclusion.
